# Experience of heart failure patients in mobilization with virtual reality: mixed methods study*

**DOI:** 10.1590/1980-220X-REEUSP-2025-0114en

**Published:** 2025-09-15

**Authors:** Larissa Gussatschenko Caballero, Iasmin Borges Fraga, Carlos Eduardo Maciel Tremea, Gabrielle Perin, Janaína dos Santos Prates, Pedro Dal Lago, João Lucas Campos de Oliveira, Eneida Rejane Rabelo-Silva

**Affiliations:** 1 Universidade Federal do Rio Grande do Sul, Hospital de Clínicas de Porto Alegre, Programa de Pós-Graduação em Enfermagem, Porto Alegre, RS, Brazil.; 2 Universidade Federal do Rio Grande do Sul, Programa de Pós-Graduação em Cardiologia e Ciências Cardiovasculares, Porto Alegre, RS, Brazil.; 3 Universidade Federal de Ciências da Saúde de Porto Alegre, Departamento de Fisioterapia, Porto Alegre, RS, Brazil.; 4 Universidade Federal do Rio Grande do Sul, Escola de Enfermagem, Porto Alegre, RS, Brazil.

**Keywords:** Patient Satisfaction, Heart Failure, Critical Care, Virtual Reality

## Abstract

**Objective:**

To compare the effects of up to three sessions of an early mobilization protocol combined with immersive virtual reality (VR) *versus* early mobilization alone in the experience of patients with acutely decompensated heart failure.

**Methods:**

Complex mixed methods study with adult patients admitted to the intensive care unit. The Intervention Group (IG) underwent mobilization combined with immersive virtual reality and the Control Group (CG), isolated mobilization. The tool Net Promoter Score (NPS) and the Likert scale were used to assess patients’ experience. In the qualitative approach, interviews were conducted with open questions.

**Results:**

Sixty patients participated in the study, 44 (73.3%) men, mean age 59.7 ± 12.2. The procedure was recommended by 23 (76.7%) in the IG and by 24 (80%) in the CG. The mobilization experience was classified as good or excellent by 28 (93.3%) in the IG and 26 (83.7%) in the CG. Qualitative analyses revealed three categories: “Psychological Effects”, “Physical Performance” and “Innovation”.

**Conclusion:**

Combining virtual reality with early mobilization provided a more positive experience for patients. *ClinicalTrials.gov* with number NCT05596292.

## INTRODUCTION

Early mobilization, as an adjunct in the treatment of patients with acutely decompensated heart failure, is highlighted for reducing the harmful effects of being confined to a hospital bed. This approach, which includes adapted exercises, physiotherapy, and activities promoting physical function, helps to mitigate the deterioration of these individuals’ functional capacity^(1,2,3)^. Scientific evidence shows that implementing early mobilization in intensive care units accelerates recovery, improves clinical outcomes, and significantly contributes to reducing hospital stays^(1,4,5,6)^. In addition to its already consolidated benefits, early mobilization also has a positive impact on patients’ psychological sphere, helping them to cope with anxiety and depression—comorbidities frequently associated with prolonged hospital stays^(7,8)^.

However, motivation and adherence to early mobilization can be challenging in patients with decompensated heart failure, due to fatigue, dyspnea, and other comorbidities^(9)^. Recent studies have consistently demonstrated the benefits of VR in this context, evidencing its ability to significantly increase patients’ motivation to engage in therapeutic exercises, while also improving their tolerance to these activities^(10)^. Additionally, immersion in interactive virtual environments has been shown to be effective in reducing the perception of pain and anxiety levels associated with medical procedures and the intensive care unit experience itself^(11)^. By providing an immersive and potentially playful experience, VR can shift the focus away from physical and emotional discomfort, creating an environment more conducive to active participation in rehabilitation and, consequently, optimizing patients’ functional recovery^(12)^.

Despite the promising benefits of VR already observed in several rehabilitation contexts and in the management of pain and anxiety, its specific application in conjunction with the early mobilization of patients with heart failure in intensive care units is still little explored. Given this gap and the potential synergy between the effects of early mobilization and the immersive experience provided by VR, the present study aimed to compare the effects of up to three sessions of an early mobilization protocol associated with immersive VR versus early mobilization alone, in the experience of patients with acutely decompensated heart failure, admitted to an intensive care unit.

## METHOD

This is a complex mixed methods study, nested within a randomized clinical trial. The integration of quantitative and qualitative data occurred in three distinct phases: in the planning stage; in the data collection and analysis phase, in which the methods were interconnected; and, finally, in the results interpretation stage, carried out through a narrative combined with visual displays—joint display. Data from the quantitative phase were used to define the criteria for the qualitative analysis of the study. Theorizing was implicit, and the methodological emphasis was even: QUANT + QUAL. The triangulation of data in this research was carried out through the convergence and complementation of the findings of the mixed methods. Quantitative data supported qualitative interpretations, with the aim of deepening the investigation and providing a more comprehensive understanding of patients’ experience with early mobilization^(13)^.

The quantitative approach was conducted through a single- center, parallel, randomized clinical trial, with blinding in the collection of outcomes. Randomization was simple, in a 1:1 ratio, and carried out by a researcher through the electronic platform Research Electronic Data Capture® (REDCap®). After recording all sample characterization variables and baseline study parameters, patients were randomly allocated into two groups: Intervention group – early mobilization protocol associated with the use of immersive VR; Control group – early mobilization protocol alone.

The previously published study protocol^(14)^ included three stages of exercises using a cycle ergometer for upper and lower limbs, sitting, standing, and walking. Session duration varied between 10 and 20 minutes, with progression adjusted to individual tolerance, based on patient perception and vital signs. IG participants performed exercises associated with the use of immersive VR glasses compatible with smartphones. During the execution, an interactive 360-degree video was displayed, allowing the patient to explore different angles of a virtual environment. Headphones were used to minimize external sound interference and provide a more immersive experience.

In the qualitative segment of the research, interviews with open questions were conducted, aimed at deepening the perceptions of participants from both groups about the mobilization protocol to which they were subjected. The interviews took place immediately after the completion of the interventions and sought to explore the scores assigned on the Net Promoter Score and Likert scales, focusing on the recommendation of mobilization and personal experience during the procedure.

The qualitative analysis of the interviews was intentionally directed at participants classified as “promoters” on the scale Net Promoter Score and who rated their experience as “good” or “excellent” on the Likert scale. This choice is justified by the fact that these individuals tend to present more detailed perceptions about the practices and conditions that favor a positive experience.

### Study Location and Collection Period

The study was developed in an intensive care unit (ICU) of a highly complex public university hospital, certified with international accreditation by the Joint Commission International. The data collection period was from January 2023 to January 2024.

### Population and Sample Definition

Patients admitted to the ICU with a diagnosis of acutely decompensated heart failure recorded in their medical records, aged 18 years or older, lucid, oriented, and coherent were considered eligible. The sample size was estimated based on the detection of differences in the mean sensation of dyspnea, measured by the Borg scale^(15)^. A variation of 1 point on the scale was considered a clinically relevant difference. The estimate was performed using the tool *PSS Health*, online version^(16)^, assuming a statistical power of 80%, significance level of 5%, and standard deviation of 1.6 points^(17,18)^. As a result, a total sample size of 54 subjects was calculated. At the end of the study, 60 patients participated.

### Study Variables

Sociodemographic and clinical variables included: age, sex, education, occupation, history of hospitalizations (current and previous), as well as information regarding the underlying pathology, comorbidities, and use of medications. The risk of falls was assessed using the Severo-Almeida-Kuchenbecker (SAK) scale^(19)^. Functional independence was measured using the Barthel Scale^(20)^. The sensation of dyspnea was assessed before and after each mobilization session, in both groups, using the Borg Scale^(15)^.

Additionally, the procedure recommendability and patient’s experience were investigated using the Net Promoter Score and the Likert Scale. The NPS score followed the tool original categorization: detractors (scores 0 to 6), passives (scores 7 and 8), and promoters (scores 9 and 10)^(21,22)^. The question applied for this evaluation was: “Using a verbal numerical scale from 0 to 10, where 0 means ‘never’ and 10 means ‘always’, how likely is it that you would recommend this mobilization procedure to another patient, friend, or colleague who may need it? Could you describe the reason for your grade?”

The participants’ experience was assessed using a Likert Scale with five subgroups, classifying the experience from very bad to excellent, with the following correspondences: 0 (very bad); 1 to 3 (bad); 4 and 5 (good); 6 to 7 (very good); 9 to 10 (excellent). The corresponding question was: “Using the verbal numerical scale from 0 to 10, where 0 means ‘the worst/terrible experience possible’ and 10 means ‘the best/excellent experience possible’, how would you rate your experience during the mobilization carried out? What is the most important reason for you to have given this rating to this procedure?”

### Data Analysis and Treatment

The database was built on the REDCap® platform. Quantitative data were analyzed using the statistical software SPSS®, version 28.0 for Windows®. Data normality was verified using the Shapiro-Wilk test. According to the distribution of variables, quantitative data were described by mean and standard deviation or, in case of non-normal distribution, by median and interquartile ranges. Categorical data were expressed in absolute and relative frequency. For the comparative analysis of variables between moments and between groups simultaneously, the Generalized Estimating Equations (GEE) model was applied. The comparison of means between groups was performed using Student’s t-test. In cases where there was data asymmetry, the Mann-Whitney test was used. To compare proportions, Pearson’s chi-square test or Fisher’s exact test were used, depending on suitability. A p value < 0.05 (two-tailed) was adopted as statistically significant. All analyses were conducted according to the intention-to-treat principle. Qualitative information, coming from open responses to questions linked to the Net Promoter Score and the Likert Scale, was collected through audio recordings and transcribed in full for the REDCap® platform. The analysis of this information was conducted based on Content Analysis^(23)^ through an exhaustive process of thorough reading of the participants’ speeches, characterized by a “back and forth” movement in the textual corpus, until the identification and categorization of the main emerging themes in both groups. Additionally, the resources available in the software NVivo®, version 14 for Windows®, were used to support the organization and interpretation of qualitative data.

### Ethical Aspects

The study was approved by the Research Ethics Committee of the Hospital de Clínicas de Porto Alegre, with number CAAE 62209822.7.0000.5327 and opinion number 5.619.917. All participants signed the Free and Informed Consent Form and authorized interviews recording. Participants’ speeches were recorded using an individual identifier (ID), numbered sequentially according to the order of inclusion in the study. Each quote was accompanied by a symbolic indication referring to the aspect addressed: Net Promoter Score (*) and Patient Experience (**).

## RESULTS

### Quantitative Approach

Of the 60 patients included in the study, 58 started the randomized clinical trial protocol. Two participants were excluded due to loss of initial follow-up, as they did not perform any mobilization sessions owing to worsening of their clinical condition.

Participants were predominantly male, totaling 44 (73.3%). The mean age was 59.7 ± 12.2 years, with a predominance of self-declared white individuals, corresponding to 42 (70%). The mean ejection fraction of the participants was 26% ± 12.4. The functional class of New York Heart Association (NYHA), in class III, was observed in 15 (50%) patients in the intervention group and in 14 (46.7%) in the control group. The Charlson Index indicated similarity between the groups regarding the presence of comorbidities, with a median of 3 (interquartile range: 2–5). Similarly, the risk of falls and functional capacity also did not show significant differences between the groups.

The complete data regarding the sample characteristics is described in Table 1.

**Table 1:** Sample characteristics – Porto Alegre, RS, Brazil, 2025.

Variables	All (n = 60) n (%)	IG (n = 30) n (%)	CG (n = 30) n (%)	p value
**Age (years)**	59.7 ± 12.2	62.0 ± 11.2	57.4 ± 12.8	0.144†
**Age range‡**	–	–	–	0.438§
18 – 59 years old	28 (26.9)	12 (40.0)	16 (53.3)	–
≥ 60 years	32 (53.3)	18 (60.0)	14 (46.7)	–
**Sex (Male)**	44 (73.3)	22 (73.3)	22 (73.3)	1,000§
**Ethnicity (White)‡**	42 (70.0)	18 (60.0)	24 (80.0)	0.272§
**NYHA classification‡**	–	–	–	0.665§
I	1 (1.7)	1 (3.3)	0 (0.0)	–
II	21 (35.0)	9 (30.0)	12 (40.0)	–
III	29 (48.3)	15 (50.0)	14 (46.7)	–
IV	9 (15.0)	5 (16.7)	4 (3.8)	–
**Charlson Index** ||	3 (2 – 5)	3 (2 – 5)	3 (2 v 4)	0.574¶
Ejection Fraction*	26.7 ± 12.4	26.2 ± 11.1	27.1 ± 13.8	0.781†
**SAK scale‡**	–	–	–	0.606§
Low risk	29 (48.3)	13 (43.3)	16 (53.3)	–
Moderate risk	18 (30.0)	9 (30.0)	9 (30.0)	–
High risk	13 (21.7)	8 (26.7)	5 (16.7)	–
**Barthel Functional Independence Scale‡**	–	–	–	0.313§
Little or no disability	19 (31.7)	9 (30.0)	10 (33.3)	–
Moderate disability	33 (55.0)	13 (50.0)	18 (60.0)	–
Severe disability	8 (13.3)	6 (20.0)	2 (6.7)	–

* Value presented as mean and ± standard deviation (SD)

† Student’s t-test

‡ The numbers refer to the frequency expressed in absolute numbers and percentage (%), as in some cases there was more than one indication, the tables were considered more than once

§ Pearson’s Chi-square tests

|| Value presented as median (25th percentile - 75th percentile)

¶ Mann-Whitney test.

The evaluation of the experience with the procedure was predominantly positive in both groups, also demonstrating a favorable trend towards the recommendation. The Net Promoter Score indicated that 13 participants were classified as “passive- neutral” or “detractors”. In turn, the Likert Scale showed an even smaller number of negative evaluations, with only 6 participants classifying their experiences as “reasonable” or “bad/terrible”.

All data relating to the participants’ experience is presented in Table 2.

**Table 2:** Patient’s experience and procedure recommendation – Porto Alegre, RS, Brazil, 2025.

Variables	All (n = 60) n (%)	IG (n = 30) n (%)	CG (n = 30) n (%)	p value
**Mobilization experience**	–	–	–	0.421*
Good/excellent	54 (90.0)	28 (93.3)	26 (86.7)	–
Reasonable	3 (5.0)	0 (0.0)	3 (10.0)	–
Bad/terrible	3 (5.0)	2 (6.7)	1 (3.3)	–
**Procedure Recommendation**	–	–	–	1.000*
Promoters	47 (78.3)	23 (76.7)	24 (80.0)	–
Passive/Neutral	9 (15.0)	5 (16.7)	4 (3.8)	–
Detractors	4 (6.7)	2 (6.7)	2 (6.7)	–

* Fisher’s exact test

### Qualitative Approach

The qualitative analysis resulted in the emergence of three thematic categories: “Psychological effects”, “Physical performance” and “Innovation”, the latter being identified exclusively in the intervention group.

In the category “Psychological Effects”, the following speeches illustrate the experiences lived by the participants. Emotional and psychological reactions were observed in both groups after the mobilization; however, in the intervention group, these manifestations were more frequent. The statements of the participants in this group also revealed greater enthusiasm regarding the mobilization procedure.

Intervention Group:

It’s good for the psychological aspect and for the person.* Look, because right there at that moment I felt free, I felt like a little bird flying, you know, I forgot my problems, everything.** (ID 22)

It seems to be a therapy, it clears your mind, it gets you off the ground and goes there, to another world.* The mind is a little cleared.** (ID 31)

Because it’s a wonderful experience.* I had never been to the famous virtual world, and I couldn’t imagine what it was like, when I saw it there I was amazed.** (ID 37)

Control Group:

Because it did me a lot of good to get out of bed. * I’m much better. ** (ID 29)

It’s not a bad thing to do, it’s something that distracts you, makes you feel light*. I really like cycling, I’ve done several exercises** (ID 46)

It’s good for us to distract ourselves a little.* It’s a new start to do exercises to get out of here.** (ID 60)

The “Physical performance” category was also observed in both groups, and may be related to the expectation of clinical improvement. However, the intervention group showed greater benefit in relation to mobilization than the control group. The intervention group’s speeches emphasized aspects linked to mobility and improved quality of life during the period in which participants were subjected to the mobilization protocol.

Intervention Group:

Today I have more courage. I think I’ll walk a little more.* It improved a lot during my time in hospital.** (ID 02)

Because it’s a wonderful experience. * In fact, cycling didn’t make me tired.** (ID 37)

I found it interesting, different. We exercise and don’t feel much, right?* I liked it better.** (ID 45)

Control Group:

Because it is necessary.* It was something that brought movement.** (ID 27)

It is recommended to do the exercise.* It was good.** (ID 43)

To start rehabilitating myself little by little.* Continue doing some type of exercise to improve my health.** (ID 60)

The “Innovation” category was present exclusively in the statements of the participants in the intervention group. Despite the potential for novelty associated with hospital admission or therapeutic procedures planned for both groups, this mention was not observed among participants in the control group. The differentiation occurred exclusively in relation to the use of VR, as demonstrated in the following statements:

I’m not much of an exercise person, and with this activity, it’s quite easy to do.* The glasses experience, I’d never seen that before. I really liked it.** (ID 03)

It’s something innovative, we end up even encouraging people to look for something different.* It seems like when I watch, listen to the music, watch that video, it becomes lighter, even though I’m tired and stuff, it’s different.** (ID 30)

Very good, it helps a lot with the patient’s disposition. After the adaptation it’s cool.* It’s new, I’ve never done it before and it helps a lot.** (ID 38)

The following are the integrated results of the mixed methods analysis, culminating in the metainference of these findings, as illustrated in Chart 1:

**Chart 1:** Integration of results and metainference of the analysis – Porto Alegre, RS, Brazil, 2025.

Quantitative Data	Qualitative Data	Metainference
• Age (years)*: 59.7 ± 12.2 • Sex (Male)†: 44 (73.3%) • Ethnicity (White): 42 (70.0%) • Charlson Index‡: 3 (2 – 5) • *New York Heart Association*: 29(48.3) Class III **Risk of falls – SAK scale** ➢ 48.3% of patients with low risk **Functional independence – Barthel Scale** ➢ 55% of patients with moderate disability. **Sensation of dyspnea – Borg** ➢ 31 (51.7%) Better/same ➢ IG 16 (53.3%) ➢ CG 15 (50%) **Net Promoter Score – Procedure recommendation** ➢ 47 (78.3%) promoters ➢ IG 23 (76.7%) ➢ CG 24 (80%) **Likert Scale – Patient Experience** ➢ 54(90%) “Good/Excellent” ➢ IG 28 (93.3%) ➢ CG 26 (86.7%)	Categorization after analysis of interviews with open questions: **Psychological Effects** ➢ Intervention group reports more pronounced psychological benefits. **Physical Performance** ➢ Improvements observed in both groups, with emphasis on the intervention group. **Innovation** Innovative aspects highlighted in the intervention group.	The integration of quantitative and qualitative data reveals that the intervention studied was safe and well accepted by patients. The high recommendation rate of the procedure indicates that, even without a significant difference between the groups, the perceived benefits enhanced the patients’ experience. The qualitative analysis reinforces these findings, highlighting the subjective benefits, such as the improvement in psychological well-being and physical performance, in addition to the perception of innovation in the intervention group.

* Value presented as mean and ± standard deviation (SD).

† The numbers refer to the frequency expressed as an absolute number and percentage (%).

‡ Value presented as median (25th percentile – 75th percentile)

## DISCUSSION

This is the first study comparing the effects of early mobilization with and without the use of immersive VR in patients with acutely decompensated heart failure admitted to the intensive care unit. The indication of mobilization, measured by Net Promoter Score, was considerably high. Furthermore, 90% of the sample studied classified the intervention as “Good/Excellent” on the Likert scale.

The patients’ experience, assessed through interviews with open questions, demonstrated that, in both the intervention group and the control group, mobilization was considered satisfactory. Based on the categorization proposed from the analysis of the participants’ statements, a notable emphasis and enthusiasm was observed in the IG regarding the innovation provided by immersive VR.

Patients with acutely decompensated heart failure represent a significant clinical challenge in intensive care units, especially due to the physical and psychological limitations resulting from repeated hospitalizations^(2)^. Bed rest, although often necessary for clinical stabilization, can lead to several complications, including sarcopenia, increased frailty, reduced tolerance to physical exertion, as well as anxiety and depression, negatively impacting the quality of life of these patients^(3,7)^.

In the context of intensive care, early mobilization is widely recognized for its proven clinical benefits^(8,9,24)^. However, despite the efforts of the scientific community to improve the quality of care, patient motivation remains one of the main challenges in the routine of intensive care units. The severity of the clinical condition is a determining factor, especially in patients who require complex treatments, such as the use of vasoactive drugs—a common situation in acutely decompensated heart failure^(6)^. Thus, the search for strategies that promote greater patient adherence to treatments represents a strong stimulus for clinical research in this field.

The insertion of innovative technologies, such as VR, in the healthcare context, has demonstrated remarkable potential to enrich the patient’s experience and improve clinical outcomes. The effectiveness of immersive VR was demonstrated in a study that evaluated its effect, compared to conventional treatment, in 106 hospitalized patients with chronic obstructive pulmonary disease. The results of the groups that used non-immersive VR were superior to those of the group undergoing conventional treatment, suggesting that non-immersive VR, alone or as a complement to a traditional rehabilitation program, can enhance the physical fitness of these patients^(25)^. This immersive VR strategy has also demonstrated, in healthy individuals, potential benefits in increasing exercise tolerance, possibly related to adjustments in the autonomic nervous system activity that favor parasympathetic predominance^(10)^.

In the specific context of heart failure, immersive VR has been explored as an ancillary method in the treatment of hospitalized patients with this condition. A randomized clinical trial evaluated the impact of immersive VR, compared to 2D images, in mitigating pain and improving quality of life in 88 individuals with severe heart failure. Analysis of the results revealed that participants treated with immersive VR (n = 52) showed a significant reduction in pain intensity compared to the control group (n = 36), although no substantial improvements in quality of life were observed in either group^(26)^. These findings suggest that immersive VR may constitute an effective non- pharmacological alternative for pain management in patients with severe heart failure.

In the present research, the integration of quantitative data from Net Promoter Score and the Likert scale, together with qualitative analyses, revealed benefits of early mobilization associated with the use of immersive VR. Although the difference between the IG and CG did not reach statistical significance, this absence can be attributed to factors such as the effectiveness already consolidated in the literature of hospitalization in a critical environment for the immediate start of symptomatic treatment of heart failure, associated with early mobilization. Still, the qualitative analysis of the responses revealed that IG participants demonstrated greater enthusiasm and appreciation for the innovation provided by immersive VR. This divergence between quantitative and qualitative findings highlights the importance of recognizing the complexity of the patient’s experience, which may not be fully captured by quantitative measures alone. This finding reinforces the need to interpret quantitative results in conjunction with qualitative data, which offer a deeper understanding of patients’ experiences.

The joint exploration of the data obtained in this study indicates that, although not conclusive, the use of immersive VR in treatment can reduce the feeling of mandatory exercise, providing a more pleasant and welcoming therapeutic journey. Qualitative assessments reinforce patients’ appreciation of VR, with responses from the intervention group standing out for their emphasis and detail of perceived benefits. This illustrates the value attributed by patients to this innovative therapeutic approach and highlights their active participation as co-producers of their own care.

In this regard, the valuation of the patient’s experience has emerged as a fundamental pillar for improving the quality of care in health services, by indicating that positive experiences during hospitalization are not restricted to favorable clinical outcomes^(27)^. In this research, the results demonstrate that the use of VR as an adjuvant resource had a positive impact on the experience of patients in intensive care environments. This technology appears to have alleviated the feeling of confinement in the hospital environment, helping to transform the treatment setting into a less intimidating and more welcoming space. These insights are essential to drive improvements in institutional practices, procedures, and policies, promoting a more integrated approach to care that is centered on patient needs.

The positive experience provided by VR contributes to patients’ well-being, promoting their autonomy and increasing the feeling of control over their clinical condition. Current literature has expanded the debate on how co-production of patient experience contributes significantly to improving the quality, safety, efficiency and outcomes of health care, adding value to care practices^(28)^.

Although the study presents promising results, it is important to recognize some limitations. Overcrowding in intensive care units, with consequent delays in releasing beds, may have compromised patients’ full participation in the mobilization protocol. Another factor to consider is the early discharge of some patients, which interrupted the continuity of the sessions and hindered the completion of the three-day protocol. Furthermore, as this is a study that associated a qualitative approach to its methodology, the results are subject to the subjectivity of individual perceptions, which may limit the generalization of the findings to other populations or clinical contexts.

However, the use of VR in clinical practice, especially in the nursing context, represents a valuable opportunity to improve the patient’s experience during hospitalization. By incorporating this technology, professionals can provide more personalized care, promoting patient satisfaction and encouraging their active participation in the therapeutic process.

## CONCLUSION

The main results of this study, evidenced by qualitative analyses, indicate that the innovation proposed by the intervention was valued by the IG participants. The results demonstrate that the combination of early mobilization with the use of immersive VR contributed to enriching the experience of patients with acutely decompensated heart failure admitted to intensive care units.

## Data Availability

The full dataset supporting the findings of this study is available upon request from the corresponding author, Eneida Rejane Rabelo da Silva. The dataset is not publicly available because it includes information related to additional, unpublished research that partially shares the same database.
