# Supplementary material for: Experience of heart failure patients in mobilization with virtual reality: mixed methods study
Source: Rev Esc Enferm USP. 2025 Sep 15;59:e20250114. doi: 10.1590/1980-220X-REEUSP-2025-0114en (PMC12439534; doi:10.1590/1980-220X-REEUSP-2025-0114en)
Supplement: Translation

# Translation: Experiência de pacientes com insuficiência cardíaca em mobilização com realidade virtual: estudo de métodos mistos*

**DOI:** 10.1590/1980-220X-REEUSP-2025-0114pt

**Authors:** Larissa Gussatschenko Caballero, Iasmin Borges Fraga, Carlos Eduardo Maciel Tremea, Gabrielle Perin, Janaína dos Santos Prates, Pedro Dal Lago, João Lucas Campos de Oliveira, Eneida Rejane Rabelo-Silva

**Affiliations:** 1 Universidade Federal do Rio Grande do Sul, Hospital de Clínicas de Porto Alegre, Programa de Pós-Graduação em Enfermagem, Porto Alegre, RS, Brasil.; 2 Universidade Federal do Rio Grande do Sul, Programa de Pós-Graduação em Cardiologia e Ciências Cardiovasculares, Porto Alegre, RS, Brasil.; 3 Universidade Federal de Ciências da Saúde de Porto Alegre, Departamento de Fisioterapia, Porto Alegre, RS, Brasil.; 4 Universidade Federal do Rio Grande do Sul, Escola de Enfermagem, Porto Alegre, RS, Brasil.

**Keywords:** Satisfação do paciente, Insuficiência cardíaca, Cuidados Críticos, Realidade virtual

## Abstract

**Objetivo:**

Comparar efeitos de até três sessões de um protocolo de mobilização precoce combinada com realidade virtual (RV) imersiva *versus* mobilização precoce isolada na experiência de pacientes com insuficiência cardíaca agudamente descompensada.

**Métodos:**

Estudo de métodos mistos complexo, com pacientes adultos internados na unidade de tratamento intensivo. O Grupo Intervenção (GI) realizou a mobilização combinada com realidade virtual imersiva e o Grupo Controle (GC), mobilização isolada. A ferramenta *Net Promoter Score* (NPS) e a escala Likert foram utilizadas para avaliar a experiência dos pacientes. Na abordagem qualitativa, foram realizadas entrevistas com perguntas abertas.

**Resultados:**

Participaram do estudo 60 pacientes, 44 (73,3%) homens, média de idade 59,7 ± 12,2. O procedimento foi recomendado por 23 (76,7%) no GI e por 24 (80%) no GC. A experiência de mobilização foi classificada como boa ou excelente por 28 (93,3%) no GI e 26 (83,7%) no GC. As análises qualitativas revelaram três categorias: “Efeitos Psicológicos”, “Desempenho Físico” e “Inovação”.

**Conclusão:**

A combinação da realidade virtual à mobilização precoce proporcionou uma experiência mais positiva aos pacientes. *ClinicalTrials.gov* sob número NCT05596292.

## INTRODUÇÃO

A mobilização precoce, como coadjuvante no tratamento de pacientes com insuficiência cardíaca agudamente descompensada, ganha destaque por reduzir os efeitos prejudiciais da restrição ao leito hospitalar. Essa abordagem, que inclui exercícios adaptados, fisioterapia e atividades que promovem a função física, contribui para mitigar a deterioração da capacidade funcional desses indivíduos^(1–3)^. Evidências científicas demonstram que a implementação da mobilização precoce em unidades de terapia intensiva acelera a recuperação, melhora os desfechos clínicos e contribui significativamente para a redução do tempo de internação^(1,4–6)^. Além de seus benefícios já consolidados, a mobilização precoce também exerce um impacto positivo na esfera psicológica dos pacientes, auxiliando no enfrentamento da ansiedade e da depressão—comorbidades frequentemente associadas a internações hospitalares prolongadas^(7,8)^.

No entanto, a motivação e a adesão à mobilização precoce podem ser desafiadoras em pacientes com insuficiência cardíaca descompensada, devido à fadiga, dispneia e outras comorbidades^(9)^. Estudos recentes têm demonstrado consistentemente os benefícios da RV nesse contexto, evidenciando sua capacidade de aumentar significativamente a motivação dos pacientes para se engajarem em exercícios terapêuticos, ao mesmo tempo em que melhora sua tolerância a essas atividades^(10)^. Adicionalmente, a imersão em ambientes virtuais interativos tem se mostrado eficaz na redução da percepção de dor e nos níveis de ansiedade associados a procedimentos médicos e à própria experiência na unidade de terapia intensiva^(11)^. Ao oferecer uma experiência envolvente e potencialmente lúdica, a RV pode desviar o foco do desconforto físico e emocional, criando um ambiente mais propício à participação ativa na reabilitação e, consequentemente, otimizando a recuperação funcional dos pacientes^(12)^.

Apesar dos promissores benefícios da RV já observados em diversos contextos de reabilitação e no manejo da dor e da ansiedade, sua aplicação específica em conjunto com a mobilização precoce de pacientes com insuficiência cardíaca em unidades de terapia intensiva ainda é pouco explorada. Diante dessa lacuna e da potencial sinergia entre os efeitos da mobilização precoce e a experiência imersiva proporcionada pela RV, o presente estudo teve como objetivo comparar os efeitos de até três sessões de um protocolo de mobilização precoce associada à RV imersiva versus a mobilização precoce isolada, na experiência de pacientes com insuficiência cardíaca agudamente descompensada, internados em uma unidade de cuidados intensivos.

## MÉTODO

Trata-se de um estudo de métodos mistos do tipo complexo, aninhado a um ensaio clínico randomizado. A integração dos dados quantitativos e qualitativos ocorreu em três fases distintas: na etapa de planejamento; na fase de coleta e análise de dados, em que se deu a interconexão dos métodos; e, por fim, no estágio de interpretação dos resultados, realizado por meio de uma narrativa combinada com exibições visuais—*joint display*. Os dados oriundos da fase quantitativa foram utilizados para definir os critérios da análise qualitativa do estudo. A teorização foi implícita, e a ênfase metodológica foi equânime: QUANT + QUAL. A triangulação dos dados nesta pesquisa foi realizada por meio da convergência e complementação dos achados dos métodos mistos. Os dados quantitativos sustentaram as interpretações qualitativas, com o objetivo de aprofundar a investigação e proporcionar uma compreensão mais abrangente da experiência dos pacientes com a mobilização precoce^(13)^.

A abordagem quantitativa foi conduzida por meio de um ensaio clínico randomizado, paralelo, de centro único, com cegamento na coleta dos desfechos. A randomização foi simples, na proporção 1:1, e realizada por um pesquisador por meio da plataforma eletrônica *Research Electronic Data Capture*® (REDCap®). Após o registro de todas as variáveis de caracterização da amostra e dos parâmetros basais do estudo, os pacientes foram alocados aleatoriamente em dois grupos: Grupo intervenção – protocolo de mobilização precoce associado ao uso de RV imersiva; Grupo controle – protocolo de mobilização precoce isolado.

O protocolo do estudo, previamente publicado^(14)^, incluiu três etapas de exercícios com o uso de cicloergômetro para membros superiores e inferiores, sedestação, ortostatismo e deambulação. A duração das sessões variou entre 10 e 20 minutos, com progressão ajustada à tolerância individual, baseada na percepção do paciente e nos sinais vitais. Os participantes do GI realizaram os exercícios associados ao uso de óculos de RV imersiva compatíveis com *smartphones*. Durante a execução, foi exibido um vídeo interativo em 360 graus, permitindo ao paciente explorar diferentes ângulos de um ambiente virtual. Fones de ouvido foram utilizados para minimizar interferências sonoras externas e proporcionar uma experiência mais imersiva.

No segmento qualitativo da pesquisa, foram realizadas entrevistas com perguntas abertas, com o objetivo de aprofundar as percepções dos participantes de ambos os grupos sobre o protocolo de mobilização ao qual foram submetidos. As entrevistas ocorreram imediatamente após a conclusão das intervenções e buscaram explorar as pontuações atribuídas nas escalas *Net Promoter Score* e Likert, com foco na recomendação da mobilização e na experiência pessoal durante o procedimento.

A análise qualitativa das entrevistas foi intencionalmente direcionada aos participantes classificados como “promotores” na escala *Net Promoter Score* e que avaliaram sua experiência como “boa” ou “excelente” na escala Likert. Essa escolha justifica-se pelo fato de que esses indivíduos tendem a apresentar percepções mais detalhadas sobre as práticas e condições que favorecem uma experiência positiva.

### Local do Estudo e Período de Coleta

O estudo foi desenvolvido em uma unidade de terapia intensiva (UTI) de um hospital público universitário de alta complexidade, certificado com acreditação internacional pela *Joint Commission International*. O período de coleta de dados compreendeu janeiro de 2023 a janeiro de 2024.

### População e Definição da Amostra

Foram considerados elegíveis os pacientes admitidos na UTI com diagnóstico de insuficiência cardíaca agudamente descompensada registrado em prontuário, com idade igual ou superior a 18 anos, lúcidos, orientados e coerentes. O tamanho da amostra foi estimado com base na detecção de diferenças na média da sensação de dispneia, mensurada pela escala de Borg^(15)^. Considerou-se como diferença clinicamente relevante a variação de 1 ponto na escala. A estimativa foi realizada utilizando a ferramenta *PSS Health*, versão online^(16)^, assumindo um poder estatístico de 80%, nível de significância de 5% e desvio padrão de 1,6 ponto^(17,18)^. Como resultado, foi calculado um tamanho amostral total de 54 sujeitos. Ao final do estudo, 60 pacientes participaram da pesquisa.

### Variáveis do Estudo

As variáveis sociodemográficas e clínicas incluíram: idade, sexo, escolaridade, ocupação, histórico de internações (atuais e prévias), além de informações relativas à patologia de base, comorbidades e uso de medicamentos. O risco de quedas foi avaliado por meio da escala Severo-Almeida-Kuchenbecker (SAK)^(19)^. A independência funcional foi mensurada utilizando a Escala de Barthel^(20)^. A sensação de dispneia foi avaliada antes e após cada sessão de mobilização, em ambos os grupos, por meio da Escala de Borg^(15)^.

Adicionalmente, o potencial de recomendação do procedimento e a experiência dos pacientes foram investigados utilizando-se o *Net Promoter Score* e a Escala de Likert. A pontuação do NPS seguiu a categorização original da ferramenta: detratores (notas de 0 a 6), neutros (notas 7 e 8) e promotores (notas 9 e 10)^(21,22)^. A pergunta aplicada para essa avaliação foi: “Utilizando a escala numérica verbal de 0 a 10, em que 0 significa ‘nunca’ e 10 significa ‘sempre’, qual a probabilidade de o(a) senhor(a) recomendar este procedimento de mobilização para outro paciente, amigo ou colega que venha a necessitar dele? O(a) senhor(a) poderia descrever o motivo da sua nota?”

A experiência dos participantes foi avaliada por meio de uma Escala de Likert com cinco subgrupos, classificando a experiência de péssima a excelente, com as seguintes correspondências: 0 (péssima); 1 a 3 (ruim); 4 e 5 (boa); 6 a 7 (muito boa); 9 a 10 (excelente). A pergunta correspondente foi: “Utilizando a escala numérica verbal de 0 a 10, em que 0 significa ‘a pior/péssima experiência possível’ e 10 significa ‘a melhor/excelente experiência possível’, como o(a) senhor(a) classifica a sua experiência durante a mobilização que foi realizada? Qual o motivo mais importante para o(a) senhor(a) ter atribuído essa nota a esse procedimento?”

### Tratamento e Análise dos Dados

O banco de dados foi construído na plataforma REDCap®. Os dados quantitativos foram analisados utilizando o *software* estatístico SPSS®, versão 28.0 para Windows®. A normalidade dos dados foi verificada por meio do teste de Shapiro-Wilk. Conforme a distribuição das variáveis, os dados quantitativos foram descritos por média e desvio padrão ou, em caso de distribuição não normal, por mediana e intervalos interquartílicos. Os dados categóricos foram expressos em frequência absoluta e relativa. Para a análise comparativa das variáveis entre os momentos e entre os grupos de forma simultânea, foi aplicado o modelo de Equações de Estimativas Generalizadas (GEE). A comparação de médias entre os grupos foi realizada com o teste t de Student. Nos casos em que houve assimetria dos dados, utilizou-se o teste de Mann-Whitney. Para a comparação de proporções, foram empregados os testes qui-quadrado de Pearson ou exato de Fisher, conforme a adequação. Adotou-se um valor de p < 0,05 (bicaudal) como estatisticamente significativo. Todas as análises foram conduzidas de acordo com o princípio de intenção de tratar. As informações qualitativas, provenientes das respostas abertas às perguntas vinculadas ao *Net Promoter Score* e à Escala de Likert, foram coletadas por meio de gravações de áudio e transcritas na íntegra para a plataforma REDCap®. A análise dessas informações foi conduzida com base na Análise de Conteúdo^(23)^, a partir de um processo exaustivo de leitura flutuante das falas dos participantes, caracterizado por um movimento de “ida e volta” no corpus textual, até a identificação e categorização dos principais temas emergentes em ambos os grupos. Adicionalmente, foram utilizados os recursos disponíveis no *software* NVivo®, versão 14 para *Windows*®, para apoio à organização e interpretação dos dados qualitativos.

### Aspectos Éticos

O estudo foi aprovado pelo Comitê de Ética em Pesquisa do Hospital de Clínicas de Porto Alegre, sob o número CAAE 62209822.7.0000.5327 e parecer nº 5.619.917. Todos os participantes assinaram o Termo de Consentimento Livre e Esclarecido (TCLE), bem como autorizaram a gravação de suas entrevistas. Os discursos dos participantes foram registrados utilizando um identificador individual (ID), numerado sequencialmente conforme a ordem de inclusão no estudo. Cada citação foi acompanhada de uma indicação simbólica referente ao aspecto abordado: *Net Promoter Score* (*) e Experiência do Paciente (**).

## RESULTADOS

### Abordagem Quantitativa

Dos 60 pacientes incluídos no estudo, 58 iniciaram o protocolo do ensaio clínico randomizado. Dois participantes foram excluídos por configurarem perda de seguimento inicial, uma vez que não realizaram nenhuma sessão de mobilização devido à piora da condição clínica.

Os participantes eram predominantemente do sexo masculino, totalizando 44 (73,3%). A média de idade foi de 59,7 ± 12,2 anos, com predominância de indivíduos autodeclarados brancos, correspondendo a 42 (70%). A fração de ejeção média dos participantes foi de 26% ± 12,4. A classe funcional da *New York Heart Association* (NYHA), na classe III, foi observada em 15 (50%) pacientes do grupo intervenção e em 14 (46,7%) do grupo controle. O Índice de Charlson indicou similaridade entre os grupos quanto à presença de comorbidades, com mediana de 3 (intervalo interquartílico: 2–5). De maneira semelhante, o risco de quedas e a capacidade funcional também não apresentaram diferenças significativas entre os grupos.

A integralidade dos dados referentes às características da amostra encontra-se descrita na Tabela 1.

**Tabela 1:** Características da amostra – Porto Alegre, RS, Brasil, 2025.

Variáveis	Todos (n = 60) n (%)	GI (n = 30) n (%)	GC (n = 30) n (%)	Valor P
Idade (anos)*	59,7 ± 12,2	62,0 ± 11,2	57,4 ± 12,8	0,144†
**Faixa Etária‡**	–	–	–	0,438§
18 – 59 anos	28 (46,7)	12 (40,0)	16 (53,3)	–
≥ 60 anos	32 (53,3)	18 (60,0)	14 (46,7)	–
**Sexo (Masculino)‡**	44 (73,3)	22 (73,3)	22 (73,3)	1,000§
**Etnia (Branco)‡**	42 (70,0)	18 (60,0)	24 (80,0)	0,272§
**Classificação NYHA‡**	–	–	–	0,665§
I	1 (1,7)	1 (3,3)	0 (0,0)	–
II	21 (35,0)	9 (30,0)	12 (40,0)	–
III	29 (48,3)	15 (50,0)	14 (46,7)	–
IV	9 (15,0)	5 (16,7)	4 (13,3)	–
**Índice de Charlson||**	3 (2–5)	3 (2–5)	3 (2–4)	0,574¶
Fração de Ejeção*	26,7 ± 12,4	26,2 ± 11,1	27,1 ± 13,8	0,781†
**Escala de SAK‡**	–	–	–	0,606§
Baixo risco	29 (48,3)	13 (43,3)	16 (53,3)	–
Moderado risco	18 (30,0)	9 (30,0)	9 (30,0)	–
Alto risco	13 (21,7)	8 (26,7)	5 (16,7)	–
**Escala de independência funcional segundo Barthel‡**	–	–	–	0,313§
Pouca ou nenhuma incapacidade	19 (31,7)	9 (30,0)	10 (33,3)	–
Incapacidade moderada	33 (55,0)	13 (50,0)	18 (60,0)	–
Incapacidade grave	8 (13,3)	6 (20,0)	2 (6,7)	–

* Valor apresentado em média e ± desvio padrão (DP)

† Teste t-student

‡ Os números referem-se à frequência expressos em números absolutos e porcentagem (%), como em alguns casos tiveram mais de uma indicação, as mesas foram consideradas mais de uma vez

§ Testes Qui-quadrado de Pearson

|| Valor apresentado em mediana (percentil 25 - percentil 75)

¶ Teste de Mann-Whitney.

A avaliação da experiência com o procedimento foi predominantemente positiva em ambos os grupos, demonstrando também uma tendência favorável à recomendação. O *Net Promoter Score* indicou que 13 participantes foram classificados como “passivos-neutros” ou “detratores”. Por sua vez, a Escala de Likert evidenciou um número ainda menor de avaliações negativas, com apenas 6 participantes classificando suas experiências como “razoável” ou “ruim/péssima”.

A totalidade dos dados referentes à experiência dos participantes encontra-se apresentada na Tabela 2.

**Tabela 2:** Experiência do paciente e recomendação do procedimento – Porto Alegre, RS, Brasil, 2025.

Variáveis	Todos (n = 60) n (%)	GI (n = 30) n (%)	GC (n = 30) n (%)	Valor P
**Experiência mobilização**	–	–	–	0,421*
Bom/excelente	54 (90,0)	28 (93,3)	26 (86,7)	–
Razoável	3 (5,0)	0 (0,0)	3 (10,0)	–
Ruim/péssimo	3 (5,0)	2 (6,7)	1 (3,3)	–
**Recomendação procedimento**	–	–	–	1,000*
Promotores	47 (78,3)	23 (76,7)	24 (80,0)	–
Passivos/Neutros	9 (15,0)	5 (16,7)	4 (13,3)	–
Detratores	4 (6,7)	2 (6,7)	2 (6,7)	–

* Teste exato de Fisher

### Abordagem Qualitativa

A realização da análise qualitativa resultou na emergência de três categorias temáticas: “Efeitos psicológicos”, “Desempenho físico” e “Inovação”, sendo esta última identificada exclusivamente no grupo intervenção.

Na categoria “Efeitos psicológicos”, os discursos a seguir ilustram as experiências vivenciadas pelos participantes. Reações emocionais e psicológicas foram observadas em ambos os grupos após a realização da mobilização; entretanto, no grupo intervenção, essas manifestações foram mais frequentes. As falas dos participantes desse grupo também revelaram maior entusiasmo em relação à realização do procedimento de mobilização.

Grupo Intervenção:

É bom para o psicológico e para a pessoa. * Olha porque ali no momento me senti livre, parecia um passarinho voando sabe, esqueci dos problemas, de tudo. ** (ID 22)

Parece ser uma terapia, clareia a mente, sai do chão e vai para lá, para outro mundo. * A mente clareia um pouco.** (ID 31)

Porque é uma experiência maravilhosa. * Eu nunca tinha estado no famoso mundo virtual, e eu não conseguia imaginar assim como foi, quando eu vi aquilo ali eu fiquei embasbacado. ** (ID 37)

Grupo Controle:

Porque me fez muito bem sair da cama. * Tô bem melhor. ** (ID 29)

Não é uma coisa ruim de fazer, é uma coisa que te distrai, te deixa leve*. Eu gosto muito de bicicleta, eu já passei por vários exercícios** (ID 46)

É bom a gente se distrair um pouco.* É um recomeço pra fazer exercícios pra sair daqui.**(ID 60)

A categoria “Desempenho físico” também foi observada em ambos os grupos, podendo estar relacionada à expectativa de melhora clínica. No entanto, o grupo intervenção apresentou maior benefício em relação à mobilização do que o grupo controle. Os discursos do grupo intervenção enfatizaram aspectos ligados à mobilidade e à melhora da qualidade de vida durante o período em que os participantes foram submetidos ao protocolo de mobilização.

Grupo Intervenção:

Hoje eu já tenho mais coragem. Eu acho que vou caminhar um pouco a mais.* Melhorou bastante o tempo que estive internada.** (ID 02)

Porque é uma experiência maravilhosa. * Inclusive, pedalar não me deu cansaço.** (ID 37)

Achei interessante, diferente. Faz exercício e não sente muito, né.* Eu curti mais.** (ID 45)

Grupo Controle:

Porque é necessário.* Foi uma coisa que trouxe movimento.** (ID 27)

O indicado é fazer o exercício.* Foi bom.** (ID 43)

Pra começar a se reabilitar aos poucos.* Continuar fazendo algum tipo de exercício para melhorar minha saúde.** (ID 60)

A categoria “Inovação” esteve presente exclusivamente nas manifestações dos participantes do grupo intervenção. Apesar do potencial de novidade associado à internação hospitalar ou aos procedimentos terapêuticos previstos para ambos os grupos, essa menção não foi observada entre os participantes do grupo controle. A diferenciação ocorreu exclusivamente em relação ao uso da RV, como demonstrado nas falas a seguir:

Eu não sou muito de fazer exercícios, e com essa atividade, é bem tranquilo de fazer.* A experiência dos óculos, nunca tinha visto isso. Gostei muito.** (ID 03)

É uma coisa inovadora, a gente acaba até incentivando a buscar algo diferente.* Parece assim que eu olhando, escutando a música, olhando aquele vídeo, se tornou mais leve, mesmo tendo canseira e coisa e tal, é diferente.** (ID 30)

Muito bom, ajuda bastante na disposição do paciente. Depois da adaptação é legal.* É novo, nunca tinha feito e ajuda muito. ** (ID 38)

A seguir, são apresentados os resultados integrados da análise dos métodos mistos, culminando na metainferência desses achados, conforme ilustrado na Quadro 1:

**Quadro 1:** Integração dos resultados e metainferência da análise – Porto Alegre, RS, Brasil, 2025.

Dados Quantitativos	Dados Qualitativos	Meta inferência
• Idade (anos)*: 59,7 ± 12,2 • Sexo (Masculino)†: 44 (73,3%) • Etnia (Branco): 42 (70,0%) • Índice de Charlson‡: 3 (2 – 5) • *New York Heart Association*: 29(48,3) Classe III **Risco de quedas – Escala SAK** ➢ 48,3% dos pacientes com baixo risco **Independência funcional – Escala de Barthel** ➢ 55% dos pacientes com incapacidade moderada. **Sensação de dispneia – Borg** ➢ 31 (51,7%) Melhor/igual ➢ GI 16 (53,3%) ➢ GC 15 (50%) ***Net Promoter Score* – Recomendação do procedimento** ➢ 47 (78,3%) promotores ➢ GI 23 (76,7%) ➢ GC 24 (80%) **Escala Likert – Experiência dos pacientes** ➢ 54(90%) “Boas/Excelentes” ➢ GI 28 (93,3%) ➢ GC 26 (86,7%)	Categorização após análise das entrevistas com as perguntas abertas: **Efeitos Psicológicos** ➢ Grupo intervenção relata benefícios psicológicos mais acentuados. **Desempenho Físico** ➢ Melhorias observadas em ambos os grupos, com destaque para o grupo intervenção. **Inovação** Aspectos inovadores ressaltados no grupo intervenção.	A integração dos dados quantitativos e qualitativos revela que a intervenção estudada foi segura e bem aceita pelos pacientes. A alta taxa de recomendação do procedimento indica que, mesmo sem diferença significativa entre os grupos, os benefícios percebidos enalteceram a experiência dos pacientes. A análise qualitativa reforça esses achados, destacando os benefícios subjetivos, como a melhora no bem-estar psicológico e no desempenho físico, além da percepção de inovação no grupo de intervenção.

* Valor apresentado em média e ± desvio padrão (DP).

† Os números referem-se à frequência expressos em número absoluto e porcentagem (%).

‡ Valor apresentado em mediana (percentil 25 – percentil 75)

## DISCUSSÃO

Este é o primeiro estudo a comparar os efeitos da mobilização precoce com e sem o uso da RV imersiva em pacientes com insuficiência cardíaca agudamente descompensada, internados em unidade de terapia intensiva. A indicação da mobilização, medida pelo *Net Promoter Score*, foi consideravelmente alta. Além disso, 90% da amostra estudada classificou a intervenção como “Boa/Excelente” na escala Likert.

A experiência dos pacientes, avaliada por meio de entrevistas com perguntas abertas, demonstrou que, tanto no grupo intervenção quanto no grupo controle, a mobilização foi considerada satisfatória. Com base na categorização proposta a partir da análise das falas dos participantes, observou-se no GI um destaque marcante e entusiasmo em relação à inovação proporcionada pela RV imersiva.

Pacientes com insuficiência cardíaca agudamente descompensada representam um desafio clínico significativo em unidades de terapia intensiva, especialmente pelas limitações físicas e psicológicas decorrentes das repetidas internações^(2)^. A restrição ao leito, embora muitas vezes necessária para a estabilização clínica, pode acarretar diversas complicações, incluindo sarcopenia, aumento da fragilidade, redução da tolerância ao esforço físico, além de quadros de ansiedade e depressão, impactando negativamente a qualidade de vida desses pacientes^(3,7)^.

No contexto da terapia intensiva, a mobilização precoce é amplamente reconhecida pelos seus benefícios clínicos comprovados^(8,9,24)^. No entanto, apesar dos esforços da comunidade científica para aprimorar a qualidade da assistência, a motivação dos pacientes permanece como um dos principais desafios na rotina das unidades de terapia intensiva. A gravidade do quadro clínico é um fator determinante, sobretudo em pacientes que necessitam de tratamentos complexos, como o uso de drogas vasoativas, situação comum na insuficiência cardíaca agudamente descompensada^(6)^. Assim, a busca por estratégias que promovam maior adesão dos pacientes aos tratamentos representa um forte estímulo para a pesquisa clínica nesse campo.

A inserção de tecnologias inovadoras, como a RV no contexto da saúde, tem demonstrado notável potencial para enriquecer a experiência dos pacientes e melhorar os resultados clínicos. A eficácia da RV imersiva foi evidenciada em um estudo que avaliou o seu efeito, em comparação ao tratamento convencional, em 106 pacientes hospitalizados com doença pulmonar obstrutiva crônica. Os resultados dos grupos que utilizaram RV não imersiva foram superiores aos do grupo submetido ao tratamento convencional, sugerindo que a RV não imersiva, isoladamente ou como complemento a um programa tradicional de reabilitação, pode potencializar a aptidão física desses pacientes^(25)^. Essa estratégia de RV imersiva também demonstrou, em indivíduos saudáveis, benefícios potenciais no aumento da tolerância ao exercício, possivelmente relacionados a ajustes na atividade do sistema nervoso autônomo que favorecem a predominância parassimpática^(10)^.

No contexto específico da insuficiência cardíaca, a RV imersiva foi explorada como método auxiliar no tratamento de pacientes hospitalizados com essa condição. Um ensaio clínico randomizado avaliou o impacto da RV imersiva, em comparação a imagens 2D, na mitigação da dor e na melhoria da qualidade de vida de 88 indivíduos com insuficiência cardíaca grave. A análise dos resultados revelou que os participantes tratados com RV imersiva (n = 52) apresentaram uma redução significativa na intensidade da dor em relação ao grupo controle (n = 36), embora não tenham sido observadas melhorias substanciais na qualidade de vida em nenhum dos grupos^(26)^. Esses achados sugerem que a RV imersiva pode constituir uma alternativa não farmacológica eficaz para o manejo da dor em pacientes com insuficiência cardíaca grave.

Na presente pesquisa, a integração dos dados quantitativos do *Net Promoter Score* e da escala Likert, juntamente com as análises qualitativas, revelou benefícios da mobilização precoce associada ao uso da RV imersiva. Embora a diferença entre o GI e o GC não tenha alcançado significância estatística, essa ausência pode ser atribuída a fatores como a eficácia já consolidada na literatura da internação em ambiente crítico para o início imediato do tratamento sintomático da insuficiência cardíaca, associada à mobilização precoce. Ainda assim, a análise qualitativa das respostas revelou que os participantes do GI demonstraram maior entusiasmo e valorização da inovação proporcionada pela RV imersiva. Essa divergência entre os achados quantitativos e qualitativos ressalta a importância de reconhecer a complexidade da experiência do paciente, a qual pode não ser totalmente capturada por medidas quantitativas isoladas. Tal constatação reforça a necessidade de interpretar os resultados quantitativos em conjunto com os dados qualitativos, que oferecem uma compreensão mais aprofundada da vivência dos pacientes.

A exploração conjunta dos dados obtidos neste estudo indica que, embora não conclusivo, o uso da RV imersiva no tratamento pode reduzir a sensação de obrigatoriedade dos exercícios, proporcionando uma jornada terapêutica mais prazerosa e acolhedora. As avaliações qualitativas reforçam a apreciação dos pacientes pela RV, com as respostas do grupo de intervenção destacando-se pela ênfase e detalhamento dos benefícios percebidos. Isso ilustra o valor atribuído pelos pacientes a essa abordagem terapêutica inovadora e evidencia sua participação ativa como coprodutores de seus próprios cuidados.

Nesse sentido, a valorização da experiência do paciente tem emergido como um pilar fundamental para a melhoria da qualidade assistencial nos serviços de saúde, ao indicar que experiências positivas durante a internação não se restringem apenas a desfechos clínicos favoráveis^(27)^. Nesta pesquisa, os resultados demonstram que o uso da RV como recurso coadjuvante teve impacto positivo na experiência dos pacientes em ambientes de cuidados intensivos. Essa tecnologia parece ter atenuado a sensação de confinamento no ambiente hospitalar, contribuindo para transformar o cenário de tratamento em um espaço menos intimidador e mais acolhedor. Essas percepções são essenciais para impulsionar o aperfeiçoamento das práticas, procedimentos e políticas institucionais, promovendo uma abordagem de cuidado mais integrada e centrada nas necessidades dos pacientes.

A experiência positiva proporcionada pela RV contribui para o bem-estar dos pacientes, promovendo sua autonomia e aumentando a sensação de controle sobre sua condição clínica. A literatura atual tem ampliado o debate sobre como a coprodução da experiência do paciente contribui significativamente para a melhoria da qualidade, segurança, eficiência e resultados dos cuidados de saúde, agregando valor às práticas assistenciais^(28)^.

Embora o estudo apresente resultados promissores, é importante reconhecer algumas limitações. A superlotação das unidades de terapia intensiva, com consequente demora na liberação de leitos, pode ter comprometido a participação plena dos pacientes no protocolo de mobilização. Outro fator a considerar é a alta precoce de alguns pacientes, que interrompeu a continuidade das sessões e impossibilitou a conclusão do protocolo de três dias. Além disso, por se tratar de um estudo que associou uma abordagem qualitativa em sua metodologia, os resultados estão sujeitos à subjetividade das percepções individuais, o que pode limitar a generalização dos achados para outras populações ou contextos clínicos.

Entretanto, a utilização da RV na prática clínica, especialmente no contexto da enfermagem, representa uma oportunidade valiosa para aprimorar a experiência do paciente durante a hospitalização. Ao incorporar essa tecnologia, os profissionais podem oferecer um cuidado mais personalizado, promovendo a satisfação dos pacientes e incentivando sua participação ativa no processo terapêutico.

## CONCLUSÃO

Os principais resultados deste estudo, evidenciado pelas análises qualitativas, indica que a inovação proposta pela intervenção foi valorizada pelos participantes do GI. Os resultados demonstram que a combinação da mobilização precoce com o uso da RV imersiva contribuiu para enriquecer a experiência de pacientes com insuficiência cardíaca agudamente descompensada internados em unidades de terapia intensiva.

## Data Availability

Todo o conjunto de dados que dá suporte aos resultados deste estudo está disponível mediante solicitação ao autor correspondente, Eneida Rejane Rabelo da Silva. O conjunto de dados não está publicamente disponível pois inclui informações relacionadas a pesquisas adicionais ainda não publicadas, que compartilham parcialmente o mesmo banco de dados.

# Translation: Experiencia de pacientes con insuficiencia cardíaca en movilización con realidad virtual: estudio de métodos mixtos*

**DOI:** 10.1590/1980-220X-REEUSP-2025-0114es

**Authors:** Larissa Gussatschenko Caballero, Iasmin Borges Fraga, Carlos Eduardo Maciel Tremea, Gabrielle Perin, Janaína dos Santos Prates, Pedro Dal Lago, João Lucas Campos de Oliveira, Eneida Rejane Rabelo-Silva

**Affiliations:** 1 Universidade Federal do Rio Grande do Sul, Hospital de Clínicas de Porto Alegre, Programa de Pós-Graduação em Enfermagem, Porto Alegre, RS, Brasil.; 2 Universidade Federal do Rio Grande do Sul, Programa de Pós-Graduação em Cardiologia e Ciências Cardiovasculares, Porto Alegre, RS, Brasil.; 3 Universidade Federal de Ciências da Saúde de Porto Alegre, Departamento de Fisioterapia, Porto Alegre, RS, Brasil.; 4 Universidade Federal do Rio Grande do Sul, Escola de Enfermagem, Porto Alegre, RS, Brasil.

**Keywords:** Satisfacción del Paciente, Insuficiencia Cardíaca, Cuidados Críticos, Realidad Virtual

## Abstract

**Objetivo:**

Comparar los efectos de hasta tres sesiones de un protocolo de movilización temprana combinada con realidad virtual (RV) inmersiva *versus* movilización precoz sola en la experiencia de pacientes con insuficiencia cardíaca aguda descompensada.

**Métodos:**

Estudio complejo de métodos mixtos con pacientes adultos ingresados en la unidad de cuidados intensivos. El Grupo de Intervención (GI) realizó movilización combinada con realidad virtual inmersiva y el Grupo Control (GC), movilización aislada. Se utilizaron la herramienta *Net Promoter Score* (NPS) y la escala de Likert para evaluar la experiencia del paciente. En el enfoque cualitativo se realizaron entrevistas con preguntas abiertas.

**Resultados:**

Participaron en el estudio sesenta pacientes, 44 (73,3%) hombres, con una edad media de 59,7 ± 12,2. El procedimiento fue recomendado por 23 (76,7%) en el GI y por 24 (80%) en el GC. La experiencia de movilización fue clasificada como buena o excelente por 28 (93,3%) en el GI y 26 (83,7%) en el GC. Los análisis cualitativos revelaron tres categorías: “Efectos psicológicos”, “Rendimiento físico” e “Innovación”.

**Conclusión:**

La combinación de la realidad virtual con la movilización temprana proporcionó una experiencia más positiva para los pacientes. *ClinicalTrials.gov* bajo el número NCT05596292.

## INTRODUCCIÓN

La movilización temprana, como coadyuvante en el tratamiento de pacientes con insuficiencia cardíaca aguda descompensada, se destaca por reducir los efectos perjudiciales de la restricción al lecho hospitalario. Este enfoque, que incluye ejercicios adaptados, fisioterapia y actividades que promueven la función física, contribuye a mitigar el deterioro de la capacidad funcional de estos individuos^(1–3)^. Las pruebas científicas demuestran que la implementación de la movilización temprana en las unidades de terapia intensiva acelera la recuperación, mejora los resultados clínicos y contribuye significativamente a la reducción del tiempo de hospitalización^(1,4–6)^. Además de sus beneficios ya consolidados, la movilización temprana también tiene un impacto positivo en la esfera psicológica de los pacientes, ayudándoles a afrontar la ansiedad y la depresión, comorbilidades frecuentemente asociadas a hospitalizaciones prolongadas^(7,8)^.

Sin embargo, la motivación y la adherencia a la movilización temprana pueden ser un reto en pacientes con insuficiencia cardíaca descompensada, debido a la fatiga, la disnea y otras comorbilidades^(9)^. Estudios recientes han demostrado de manera consistente los beneficios de la Realidad Virtual (RV) en este contexto, evidenciando su capacidad para aumentar significativamente la motivación de los pacientes para realizar ejercicios terapéuticos, al tiempo que mejora su tolerancia a estas actividades^(10)^. Además, la inmersión en entornos virtuales interactivos ha demostrado ser eficaz para reducir la percepción del dolor y los niveles de ansiedad asociados a los procedimientos médicos y a la propia experiencia en la unidad de cuidados intensivos^(11)^. Al ofrecer una experiencia envolvente y potencialmente lúdica, la RV puede desviar la atención del malestar físico y emocional, creando un entorno más propicio para la participación activa en la rehabilitación y, en consecuencia, optimizando la recuperación funcional de los pacientes^(12)^.

A pesar de los prometedores beneficios de la RV ya observados en diversos contextos de rehabilitación y en el manejo del dolor y la ansiedad, su aplicación específica en conjunto con la movilización temprana de pacientes con insuficiencia cardíaca en unidades de terapia intensiva aún es poco explorada. Ante este vacío de conocimiento y de la sinergia potencial entre los efectos de la movilización temprana y la experiencia inmersiva proporcionada por la RV, el presente estudio tuvo como objetivo comparar los efectos de hasta tres sesiones de un protocolo de movilización temprana asociada a la RV inmersiva frente a la movilización temprana aislada, en la experiencia de pacientes con insuficiencia cardíaca aguda descompensada, ingresados en una unidad de cuidados intensivos.

## MÉTODO

Se trata de un estudio de métodos mixtos de tipo complejo, anidado en un ensayo clínico aleatorizado. La integración de los datos cuantitativos y cualitativos se realizó en tres fases distintas: en la etapa de planificación; en la fase de recolección y análisis de datos, en la que se produjo la interconexión de los métodos; y, por último, en la etapa de interpretación de los resultados, realizada mediante una narrativa combinada con exhibiciones visuales (*joint display*). Los datos obtenidos en la fase cuantitativa se utilizaron para definir los criterios de análisis cualitativo del estudio. La teorización fue implícita y el énfasis metodológico fue equitativo: CUANTI + CUALI. La triangulación de los datos en esta investigación se realizó mediante la convergencia y complementación de los hallazgos de los métodos mixtos. Los datos cuantitativos respaldaron las interpretaciones cualitativas, con el objetivo de profundizar la investigación y proporcionar una comprensión más amplia de la experiencia de los pacientes con movilización temprana^(13)^.

El enfoque cuantitativo se llevó a cabo mediante un ensayo clínico aleatorio, paralelo, de un solo centro, con enmascaramiento en la recolección de los resultados. La aleatorización fue simple, en una proporción de 1:1, y realizada por un investigador a través de la plataforma electrónica*Research Electronic Data Capture*® (REDCap®). Tras registrar todas las variables de caracterización de la muestra y los parámetros basales del estudio, los pacientes fueron asignados aleatoriamente a dos grupos: Grupo de intervención: protocolo de movilización temprana asociado al uso de RV inmersiva; Grupo de control: protocolo de movilización temprana aislada.

El protocolo del estudio, publicado previamente^(14)^, incluyó tres etapas de ejercicios con el uso de un cicloergómetro para miembros superiores e inferiores, sedestación, ortostatismo y deambulación. La duración de las sesiones varió entre 10 y 20 minutos, con una progresión ajustada a la tolerancia individual, basada en la percepción del paciente y en los signos vitales. Los participantes del GI realizaron los ejercicios asociados al uso de gafas de RV inmersiva compatibles con*smartphones*. Durante la ejecución, se proyectó un vídeo interactivo en 360 grados, lo que permitió al paciente explorar diferentes ángulos de un entorno virtual. Se utilizaron auriculares para minimizar las interferencias sonoras externas y proporcionar una experiencia más inmersiva.

En la parte cualitativa de la investigación, se realizaron entrevistas con preguntas abiertas, con el objetivo de profundizar en las percepciones de los participantes de ambos grupos sobre el protocolo de movilización al que fueron sometidos. Las entrevistas se realizaron inmediatamente después de la finalización de las intervenciones y buscaban explorar las puntuaciones atribuidas en las escalas*Net Promoter Score*y Likert, centrándose en la recomendación de la movilización y en la experiencia personal durante el procedimiento.

El análisis cualitativo de las entrevistas se dirigió intencionadamente a los participantes clasificados como «promotores» en la escala*Net Promoter Score*y que evaluaron su experiencia como «buena» o «excelente» en la escala Likert. Esta elección se justifica por el hecho de que estos individuos tienden a presentar percepciones más detalladas sobre las prácticas y condiciones que favorecen una experiencia positiva.

### Lugar del Estudio y Período de Recolección

El estudio se llevó a cabo en una unidad de terapia intensiva (UTI) de un hospital público universitario de alta complejidad, certificado con acreditación internacional por la*Joint Commission International*. El período de recolección de datos abarcó desde enero de 2023 hasta enero de 2024.

### Población y Definición de la Muestra

Se consideraron elegibles los pacientes ingresados en la UTI con diagnóstico de insuficiencia cardíaca aguda descompensada registrado en el historial clínico, con edad igual o superior a 18 años, lúcidos, orientados y coherentes. El tamaño de la muestra se estimó basándose en la detección de diferencias en la media de la sensación de disnea, medida mediante la escala de Borg^(15)^. Se consideró como diferencia clínicamente relevante la variación de 1 punto en la escala. La estimación se realizó utilizando la herramienta*PSS Health*, versión en línea^(16)^, asumiendo un poder estadístico del 80%, un nivel de significación del 5% y una desviación estándar de 1,6 puntos^(17,18)^. Como resultado, se calculó un tamaño muestral total de 54 sujetos. Al final del estudio, participaron en la investigación 60 pacientes.

### Variables del Estudio

Las variables sociodemográficas y clínicas incluyeron: edad, sexo, nivel educativo, ocupación, historial de hospitalizaciones (actuales y previas), además de información relativa a la patología base, comorbilidades y uso de medicamentos. El riesgo de caídas se evaluó mediante la escala Severo-Almeida-Kuchenbecker (SAK)^(19)^. La independencia funcional se midió utilizando la Escala de Barthel^(20)^. La sensación de disnea se evaluó antes y después de cada sesión de movilización, en ambos grupos, mediante la Escala de Borg^(15)^.

Además, se investigó el potencial de recomendación del procedimiento y la experiencia de los pacientes utilizando el*Net Promoter Score*y la Escala de Likert. La puntuación del NPS siguió la categorización original de la herramienta: detractores (puntuaciones de 0 a 6), neutrales (puntuaciones 7 y 8) y promotores (puntuaciones 9 y 10)^(21,22)^. La pregunta aplicada para esta evaluación fue: «Utilizando la escala numérica verbal del 0 al 10, en la que 0 significa «nunca» y 10 significa «siempre», ¿qué probabilidad hay de que recomiende este procedimiento de movilización a otro paciente, amigo o compañero que lo necesite? ¿Podría describir el motivo de su puntuación?».

La experiencia de los participantes se evaluó mediante una escala de Likert con cinco subgrupos, que clasificaban la experiencia de pésima a excelente, con las siguientes correspondencias: 0 (pésima); 1 a 3 (mala); 4 y 5 (buena); 6 a 7 (muy buena); 9 a 10 (excelente). La pregunta correspondiente fue: «Utilizando la escala numérica verbal del 0 al 10, en la que 0 significa «la peor/mala experiencia posible» y 10 significa «la mejor/excelente experiencia posible», ¿cómo calificaría su experiencia durante la movilización que se llevó a cabo? ¿Cuál es el motivo más importante por el que ha otorgado esa puntuación a este procedimiento?».

### Tratamiento y Análisis de Los Datos

La base de datos se construyó en la plataforma REDCap®. Los datos cuantitativos se analizaron utilizando el*software* estadístico SPSS®, versión 28.0 para Windows®. La normalidad de los datos se verificó mediante la prueba de Shapiro-Wilk. Según la distribución de las variables, los datos cuantitativos se describieron mediante la media y la desviación estándar o, en caso de distribución no normal, mediante la mediana y los intervalos intercuartílicos. Los datos categóricos se expresaron en frecuencia absoluta y relativa. Para el análisis comparativo de las variables entre los momentos y entre los grupos de forma simultánea, se aplicó el modelo de ecuaciones de estimaciones generalizadas (GEE). La comparación de medias entre los grupos se realizó con la prueba t de Student. En los casos en que hubo asimetría en los datos, se utilizó la prueba de Mann-Whitney. Para la comparación de proporciones, se emplearon las pruebas qui-cuadrado de Pearson o exacta de Fisher, según la idoneidad. Se adoptó un valor de p < 0,05 (bicaudal) como estadísticamente significativo. Todas las análisis se realizaron de acuerdo con el principio de intención de tratar. La información cualitativa, procedente de las respuestas abiertas a las preguntas relacionadas con el*Net Promoter Score*y la Escala de Likert, se recopiló mediante grabaciones de audio y se transcribió íntegramente a la plataforma REDCap®. El análisis de esta información se llevó a cabo basándose en el Análisis de Contenido^(23)^, a partir de un exhaustivo proceso de lectura flotante de las declaraciones de los participantes, caracterizado por un movimiento de «ida y vuelta» en el corpus textual, hasta la identificación y categorización de los principales temas emergentes en ambos grupos. Además, se utilizaron los recursos disponibles en el*software*NVivo®, versión 14 para*Windows*®, para apoyar la organización e interpretación de los datos cualitativos.

### Aspectos Éticos

El estudio fue aprobado por el Comité de Ética en Investigación del Hospital de Clínicas de Porto Alegre, con el número CAAE 62209822.7.0000.5327 y el dictamen n.º 5.619.917. Todos los participantes firmaron el Término de Consentimiento Libre y Esclarecido (TCLE) y autorizaron la grabación de sus entrevistas. Los discursos de los participantes se registraron utilizando un identificador individual (ID), numerado secuencialmente según el orden de inclusión en el estudio. Cada cita fue acompañada de una indicación simbólica relativa al aspecto abordado:*Net Promoter Score*(*) y Experiencia del Paciente (**).

## RESULTADOS

### Enfoque Cuantitativo

De los 60 pacientes incluidos en el estudio, 58 iniciaron el protocolo del ensayo clínico aleatorizado. Dos participantes fueron excluidos por pérdida de seguimiento inicial, ya que no realizaron ninguna sesión de movilización debido al empeoramiento de su estado clínico.

Los participantes eran predominantemente varones, con un total de 44 (73,3%). La media de edad fue de 59,7 ± 12,2 años, con predominio de individuos que se declararon blancos, lo que corresponde al 42 (70%). La fracción de eyección media de los participantes fue del 26% ± 12,4. La clase funcional de la*New York Heart Association*(NYHA), en clase III, se observó en 15 (50%) pacientes del grupo de intervención y en 14 (46,7%) del grupo de control. El índice de Charlson indicó similitud entre los grupos en cuanto a la presencia de comorbilidades, con una mediana de 3 (intervalo intercuartílico: 2–5). De manera similar, el riesgo de caídas y la capacidad funcional tampoco presentaron diferencias significativas entre los grupos.

La integridad de los datos relativos a las características de la muestra se describe en la Tabla 1.

**Tabla 1:** Características de la muestra – Porto Alegre, RS, Brasil, 2025.

Variables	Todos (n = 60) n (%)	GI (n = 30) n (%)	GC (n = 30) n (%)	Valor P
Edad (años)*	59,7 ± 12,2	62,0 ± 11,2	57,4 ± 12,8	0,144†
**Grupos de edad‡**	–	–	–	0,438§
18 – 59 años	28 (46,7)	12 (40,0)	16 (53,3)	–
≥ 60 años	32 (53,3)	18 (60,0)	14 (46,7)	–
**Sexo (Masculino)‡**	44 (73,3)	22 (73,3)	22 (73,3)	1,000§
**Etnia (Blanco)‡**	42 (70,0)	18 (60,0)	24 (80,0)	0,272§
**Clasificación NYHA‡**	–	–	–	0,665§
I	1 (1,7)	1 (3,3)	0 (0,0)	–
II	21 (35,0)	9 (30,0)	12 (40,0)	–
III	29 (48,3)	15 (50,0)	14 (46,7)	–
IV	9 (15,0)	5 (16,7)	4 (13,3)	–
Índice de Charlson||	3 (2 – 5)	3 (2 – 5)	3 (2 – 4)	0,574¶
Fracción de eyección*	26,7 ± 12,4	26,2 ± 11,1	27,1 ± 13,8	0,781†
**Escala de SAK‡**	–	–	–	0,606§
Bajo riesgo	29 (48,3)	13 (43,3)	16 (53,3)	–
Moderado riesgo	18 (30,0)	9 (30,0)	9 (30,0)	–
Alto riesgo	13 (21,7)	8 (26,7)	5 (16,7)	–
**Escala de independencia funcional de acuerdo a Barthel‡**	–	–	–	0,313§
Poca o ninguna incapacidad	19 (31,7)	9 (30,0)	10 (33,3)	–
Incapacidad moderada	33 (55,0)	13 (50,0)	18 (60,0)	–
Incapacidad grave	8 (13,3)	6 (20,0)	2 (6,7)	–

* Valor presentado en media y ± desviación estándar (DE)

† Prueba t de Student

‡ Las cifras se refieren a la frecuencia expresada en números absolutos y porcentajes (%), ya que en algunos casos había más de una indicación, las mesas se consideraron más de una vez

§ Pruebas Chi-cuadrado de Pearson

|| Valor presentado en mediana (percentil 25 - percentil 75)

¶ Prueba de Mann-Whitney.

La evaluación de la experiencia con el procedimiento fue predominantemente positiva en ambos grupos, lo que también demostró una tendencia favorable a la recomendación. El*Net Promoter Score*indicó que 13 participantes fueron clasificados como «pasivos-neutros» o «detractores». Por su parte, la escala de Likert mostró un número aún menor de evaluaciones negativas, con solo 6 participantes que calificaron sus experiencias como «razonables» o «malas/pésimas».

Todos los datos relativos a la experiencia de los participantes se presentan en la Tabla 2.

**Tabla 2:** Experiencia del paciente y recomendación del procedimiento – Porto Alegre, RS, Brasil, 2025.

Variables	Todos (n = 60) n (%)	GI (n = 30) n (%)	GC (n = 30) n (%)	Valor P
**Experiencia movilización**	–	–	–	0,421*
Buena/excelente	54 (90,0)	28 (93,3)	26 (86,7)	–
Razonable	3 (5,0)	0 (0,0)	3 (10,0)	–
Mala/pésima	3 (5,0)	2 (6,7)	1 (3,3)	–
**Recomendación procedimiento**	–	–	–	1,000*
Promotores	47 (78,3)	23 (76,7)	24 (80,0)	–
Pasivos/Neutros	9 (15,0)	5 (16,7)	4 (13,3)	–
Detractores	4 (6,7)	2 (6,7)	2 (6,7)	–

* Test exacto de Fisher

### Enfoque Cualitativo

La realización del análisis cualitativo dio como resultado la aparición de tres categorías temáticas: «Efectos psicológicos», «Rendimiento físico» e «Innovación», siendo esta última identificada exclusivamente en el grupo de intervención.

En la categoría «Efectos psicológicos», los siguientes discursos ilustran las experiencias vividas por los participantes. Se observaron reacciones emocionales y psicológicas en ambos grupos tras la movilización; sin embargo, en el grupo de intervención, estas manifestaciones fueron más frecuentes. Las declaraciones de los participantes de este grupo también revelaron un mayor entusiasmo por la realización del procedimiento de movilización.

Grupo de intervención:

Es bueno para la psicología y para la persona. * Mira, porque en ese momento me sentí libre, parecía un pájaro volando, ¿sabes?, me olvidé de los problemas, de todo. **(ID 22)

Parece una terapia, aclara la mente, sales del suelo y vas allí, a otro mundo. * La mente se aclara un poco.**(ID 31)

Porque es una experiencia maravillosa. * Nunca había estado en el famoso mundo virtual y no podía imaginarme cómo era, cuando lo vi allí me quedé boquiabierto. **(ID 37)

Grupo de control:

Porque me hizo mucho bien salir de la cama. * Estoy mucho mejor. **(ID 29)

No es algo malo, es algo que te distrae, te hace sentir ligero*. Me gusta mucho la bicicleta, ya he hecho varios ejercicios**(ID 46)

Es bueno distraerse un poco.* Es un nuevo comienzo para hacer ejercicio y salir de aquí.** (ID 60)

La categoría «Rendimiento físico» también se observó en ambos grupos, lo que podría estar relacionado con la expectativa de mejora clínica. Sin embargo, el grupo de intervención presentó mayores beneficios en relación con la movilización que el grupo de control. Los discursos del grupo de intervención enfatizaron aspectos relacionados con la movilidad y la mejora de la calidad de vida durante el período en que los participantes fueron sometidos al protocolo de movilización.

Grupo de intervención:

Hoy tengo más ánimos. Creo que voy a caminar un poco más.* Ha mejorado mucho el tiempo que he estado ingresada.** (ID 02)

Porque es una experiencia maravillosa. * Además, pedalear no me cansó.**(ID 37)

Me pareció interesante, diferente. Haces ejercicio y no se nota mucho, ¿no?* Me gustó más.**(ID 45)

Grupo de control:

Porque es necesario.* Fue algo que me aportó movimiento.** (ID 27)

Lo recomendado es hacer ejercicio.* Estuvo bien.** (ID 43)

Para empezar a rehabilitarme poco a poco.* Seguir haciendo algún tipo de ejercicio para mejorar mi salud.**(ID 60)

La categoría «Innovación» estuvo presente exclusivamente en las manifestaciones de los participantes del grupo de intervención. A pesar del potencial de novedad asociado a la hospitalización o a los procedimientos terapéuticos previstos para ambos grupos, esta mención no se observó entre los participantes del grupo de control. La diferenciación se produjo exclusivamente en relación con el uso de la RV, como se demuestra en las siguientes declaraciones:

No soy muy de hacer ejercicio, y con esta actividad es muy fácil hacerlo.* La experiencia de las gafas, nunca había visto algo así. Me gustó mucho.**(ID 03)

Es algo innovador, incluso nos anima a buscar algo diferente.* Parece que, al mirar, escuchar la música y ver ese vídeo, me siento más ligero, aunque esté cansado y tal, es diferente.**(ID 30)

Muy bueno, ayuda mucho al estado de ánimo del paciente. Después de la adaptación es genial.* Es nuevo, nunca lo había hecho y ayuda mucho.** (ID 38)

A continuación, se presentan los resultados integrados del análisis de los métodos mixtos, que culminan en la metainferencia de estos hallazgos, como se ilustra en la Cuadro 1:

**Cuadro 1:** Integración de los resultados y metainferencia del análisis – Porto Alegre, RS, Brasil, 2025.

Datos Cuantitativos	Datos Cualitativos	Meta inferencia
• Edad (años)*: 59,7 ± 12,2 • Sexo (masculino)†: 44 (73,3%) • Etnia (blanca): 42 (70,0%) • Índice de Charlson‡: 3 (2 – 5) • *New York Heart Association*: 29(48,3) Clase III **Riesgo de caídas – Escala SAK** ➢ 48,3% de los pacientes con bajo riesgo **Independencia funcional – Escala de Barthel** ➢ 55% de los pacientes con discapacidad moderada. **Sensación de disnea – Borg** ➢ 31 (51,7%) Mejor/igual ➢ GI 16 (53,3%) ➢ GC 15 (50%) ***Net Promoter Score* – Recomendación del procedimiento** ➢ 47 (78,3%) promotores ➢ GI 23 (76,7%) ➢ GC 24 (80%) **Escala Likert – Experiencia de los pacientes** ➢ 54 (90%) «Buena/Excelente» ➢ GI 28 (93,3%) ➢ GC 26 (86,7%)	Categorización tras el análisis de las entrevistas con preguntas abiertas: **Efectos psicológicos** ➢ El grupo de intervención refiere beneficios psicológicos más acusados. **Rendimiento físico** ➢ Se observan mejoras en ambos grupos, destacando el grupo de intervención. **Innovación** Se destacan aspectos innovadores en el grupo de intervención.	La integración de los datos cuantitativos y cualitativos revela que la intervención estudiada fue segura y bien aceptada por los pacientes. La alta tasa de recomendación del procedimiento indica que, aunque no hubo diferencias significativas entre los grupos, los beneficios percibidos mejoraron la experiencia de los pacientes. El análisis cualitativo refuerza estos hallazgos, destacando los beneficios subjetivos, como la mejora del bienestar psicológico y el rendimiento físico, además de la percepción de innovación en el grupo de intervención.

* Valor presentado en media y ± desviación estándar (DE).

† Las cifras se refieren a la frecuencia expresada en número absoluto y porcentaje (%).

‡ Valor presentado en mediana (percentil 25 - percentil 75)

## DISCUSIÓN

Este es el primer estudio que compara los efectos de la movilización temprana con y sin el uso de RV inmersiva en pacientes con insuficiencia cardíaca aguda descompensada ingresados en una unidad de cuidados intensivos. La indicación de la movilización, medida por el*Net Promoter Score*, fue considerablemente alta. Además, el 90% de la muestra estudiada calificó la intervención como «Buena/Excelente» en la escala Likert.

La experiencia de los pacientes, evaluada mediante entrevistas con preguntas abiertas, demostró que, tanto en el grupo de intervención como en el grupo de control, la movilización se consideró satisfactoria. Basándose en la categorización propuesta a partir del análisis de las declaraciones de los participantes, se observó en el GI un destacado entusiasmo por la innovación proporcionada por la RV inmersiva.

Los pacientes con insuficiencia cardíaca aguda descompensada representan un reto clínico significativo en las unidades de terapia intensiva, especialmente por las limitaciones físicas y psicológicas derivadas de los repetidos ingresos hospitalarios^(2)^. La restricción al lecho, aunque a menudo necesaria para la estabilización clínica, puede acarrear diversas complicaciones, como sarcopenia, aumento de la fragilidad, reducción de la tolerancia al esfuerzo físico, además de cuadros de ansiedad y depresión, lo que repercute negativamente en la calidad de vida de estos pacientes^(3,7)^.

En el contexto de la terapia intensiva, la movilización temprana es ampliamente reconocida por sus beneficios clínicos demostrados^(8,9,24)^. Sin embargo, a pesar de los esfuerzos de la comunidad científica por mejorar la calidad de la asistencia, la motivación de los pacientes sigue siendo uno de los principales retos en la rutina de las unidades de terapia intensiva. La gravedad del cuadro clínico es un factor determinante, sobre todo en pacientes que necesitan tratamientos complejos, como el uso de fármacos vasoactivos, una situación habitual en la insuficiencia cardíaca aguda descompensada^(6)^. Por lo tanto, la búsqueda de estrategias que promuevan una mayor adherencia de los pacientes a los tratamientos representa un fuerte estímulo para la investigación clínica en este campo.

La incorporación de tecnologías innovadoras, como la RV en el contexto de la salud, ha demostrado un notable potencial para enriquecer la experiencia de los pacientes y mejorar los resultados clínicos. La eficacia de la RV inmersiva se ha demostrado en un estudio que evaluó su efecto, en comparación con el tratamiento convencional, en 106 pacientes hospitalizados con enfermedad pulmonar obstructiva crónica. Los resultados de los grupos que utilizaron RV no inmersiva fueron superiores a los del grupo sometido al tratamiento convencional, lo que sugiere que la RV no inmersiva, sola o como complemento de un programa tradicional de rehabilitación, puede potenciar la aptitud física de estos pacientes^(25)^. Esta estrategia de RV inmersiva también ha demostrado, en individuos sanos, beneficios potenciales en el aumento de la tolerancia al ejercicio, posiblemente relacionados con ajustes en la actividad del sistema nervioso autónomo que favorecen el predominio parasimpático^(10)^.

En el contexto específico de la insuficiencia cardíaca, la RV inmersiva se ha explorado como método auxiliar en el tratamiento de pacientes hospitalizados con esta afección. Un ensayo clínico aleatorizado evaluó el impacto de la RV inmersiva, en comparación con imágenes 2D, en la mitigación del dolor y la mejora de la calidad de vida de 88 individuos con insuficiencia cardíaca grave. El análisis de los resultados reveló que los participantes tratados con RV inmersiva (n = 52) presentaron una reducción significativa en la intensidad del dolor en comparación con el grupo control (n = 36), aunque no se observaron mejoras sustanciales en la calidad de vida en ninguno de los grupos^(26)^. Estos hallazgos sugieren que la RV inmersiva puede constituir una alternativa no farmacológica eficaz para el manejo del dolor en pacientes con insuficiencia cardíaca grave.

En la presente investigación, la integración de los datos cuantitativos del*Net Promoter Score*y de la escala Likert, junto con los análisis cualitativos, reveló los beneficios de la movilización temprana asociada al uso de la RV inmersiva. Aunque la diferencia entre el GI y el GC no alcanzó significación estadística, esta ausencia puede atribuirse a factores como la eficacia ya consolidada en la literatura de la hospitalización en un entorno crítico para el inicio inmediato del tratamiento sintomático de la insuficiencia cardíaca, asociada a la movilización temprana. No obstante, el análisis cualitativo de las respuestas reveló que los participantes del GI mostraron un mayor entusiasmo y valoración de la innovación proporcionada por la RV inmersiva. Esta divergencia entre los hallazgos cuantitativos y cualitativos subraya la importancia de reconocer la complejidad de la experiencia del paciente, que puede no ser captada en su totalidad por medidas cuantitativas aisladas. Esta constatación refuerza la necesidad de interpretar los resultados cuantitativos junto con los datos cualitativos, que ofrecen una comprensión más profunda de la experiencia de los pacientes.

La exploración conjunta de los datos obtenidos en este estudio indica que, aunque no es concluyente, el uso de la RV inmersiva en el tratamiento puede reducir la sensación de obligatoriedad de los ejercicios, proporcionando una experiencia terapéutica más agradable y acogedora. Las evaluaciones cualitativas refuerzan la apreciación de los pacientes por la RV, con las respuestas del grupo de intervención destacando por el énfasis y el detalle de los beneficios percibidos. Esto ilustra el valor que los pacientes atribuyen a este enfoque terapéutico innovador y pone de manifiesto su participación activa como coproductores de sus propios cuidados.

En este sentido, la valoración de la experiencia del paciente ha surgido como un pilar fundamental para la mejora de la calidad asistencial en los servicios de salud, al indicar que las experiencias positivas durante la hospitalización no se limitan únicamente a los resultados clínicos favorables^(27)^. En esta investigación, los resultados demuestran que el uso de la RV como recurso coadyuvante tuvo un impacto positivo en la experiencia de los pacientes en entornos de cuidados intensivos. Esta tecnología parece haber atenuado la sensación de confinamiento en el entorno hospitalario, contribuyendo a transformar el escenario del tratamiento en un espacio menos intimidatorio y más acogedor. Estas percepciones son esenciales para impulsar la mejora de las prácticas, los procedimientos y las políticas institucionales, promoviendo un enfoque de la atención más integrado y centrado en las necesidades de los pacientes.

La experiencia positiva proporcionada por la RV contribuye al bienestar de los pacientes, promoviendo su autonomía y aumentando la sensación de control sobre su condición clínica. La literatura actual ha ampliado el debate sobre cómo la coproducción de la experiencia del paciente contribuye significativamente a la mejora de la calidad, la seguridad, la eficiencia y los resultados de la atención sanitaria, añadiendo valor a las prácticas asistenciales^(28)^.

Aunque el estudio presenta resultados prometedores, es importante reconocer algunas limitaciones. El hacinamiento en las unidades de terapia intensiva, con la consiguiente demora en la liberación de camas, puede haber comprometido la plena participación de los pacientes en el protocolo de movilización. Otro factor a tener en cuenta es el alta precoz de algunos pacientes, lo que interrumpió la continuidad de las sesiones e imposibilitó la finalización del protocolo de tres días. Además, al tratarse de un estudio que asoció un enfoque cualitativo en su metodología, los resultados están sujetos a la subjetividad de las percepciones individuales, lo que puede limitar la generalización de los hallazgos a otras poblaciones o contextos clínicos.

Sin embargo, el uso de la RV en la práctica clínica, especialmente en el contexto de la enfermería, representa una valiosa oportunidad para mejorar la experiencia del paciente durante la hospitalización. Al incorporar esta tecnología, los profesionales pueden ofrecer una atención más personalizada, promoviendo la satisfacción de los pacientes y fomentando su participación activa en el proceso terapéutico.

## CONCLUSIÓN

Los principales resultados de este estudio, evidenciados por los análisis cualitativos, indican que la innovación propuesta por la intervención fue valorada por los participantes del GI. Los resultados demuestran que la combinación de la movilización temprana con el uso de la RV inmersiva contribuyó a enriquecer la experiencia de los pacientes con insuficiencia cardíaca aguda descompensada ingresados en unidades de terapia intensiva.

## Data Availability

El conjunto de datos completo que respalda los hallazgos de este estudio está disponible previa solicitud a la autora correspondiente, Eneida Rejane Rabelo da Silva. Este conjunto de datos no está disponible públicamente porque incluye información relacionada con investigaciones adicionales no publicadas que comparten parcialmente la misma base de datos.
